# Personal, academic and stress correlates of gastroesophageal reflux disease among college students in southwestern Saudi Arabia: A cross-section study

**DOI:** 10.1016/j.amsu.2019.10.009

**Published:** 2019-10-10

**Authors:** Nabil J. Awadalla

**Affiliations:** a Department of Family and Community Medicine, College of Medicine, King Khalid University, Abha, 61421, Saudi Arabia; b Department of Community Medicine, Faculty of Medicine, Mansoura University, Mansoura, 3551, Egypt

**Keywords:** College students, Gastroesophageal reflux disease, GERD, Prevalence, Perceived stress

## Abstract

**Background:**

Gastroesophageal reflux disease (GERD) is a worldwide prevalent gastrointestinal disorder which has negative impacts on quality of life, health and economy. The aims of this study were to assess the prevalence of GERD among college students in southwestern Saudi Arabia and to evaluate its personal, academic and stress correlates.

**Materials and methods:**

Through a cross-sectional study design, a self-reported questionnaire was distributed between a representative sample of students in health and non-health care colleges in southwestern Saudi Arabia. The questionnaire included data for personal characteristics, academic study, and Arabic versions of GERD questionnaire (GerdQ) and Cohen's Perceived Stress Scale.

**Results:**

Out of 2878 studied students, GERD was reported by 28.6% and 36.6% of students in health and non-health care colleges respectively with an overall prevalence rate of 33.18%. It was associated with impacts on daily life in 17.2% of students. By multivariable regression analysis, GERD was significantly higher among males (aOR = 1.44, 95% CI:117–1.65), ex-smokers (aOR = 1.87), current smokers (aOR = 1.71), non-health care students (aOR = 1.36) and those exposed to high perceived stress (aOR = 1.30).

**Conclusion:**

GERD is a prevalent problem among college students in southwestern Saudi Arabia as it affects about one third of the students. Considering high prevalence of GERD, associated daily life impacts, young age of the studied subject and the risk of future complications, this condition could represent a challenging health and economic problem. The risk of GERD is higher among; males, smokers, former smokers, non-health care colleges students and subjects exposed to high perceived stress.

## Highlights

• GERD affects about one third of college students in southwestern Saudi Arabia.
• GERD is associated with daily life impacts in about one fifth of the students.
• GERD could represent a challenging health and economic problem.
• GERD is significantly associated with high perceived stress.
• Risk of GERD is higher among male students, smokers and non-health care students.

## 1 Introduction

Gastroesophageal reflux disease (GERD) is a chronic disease caused by reflux of stomach contents into the esophagus. It is accompanied by several unpleasant symptoms and may restrict personal daily activity [1]. GERD is one of the most common gastrointestinal problems worldwide, with negative impacts on quality of life, health and economy [2]. According to population-based studies, the worldwide prevalence rates of GERD generally range from 10 to 30% in Europe, America and Middle East populations. Only East Asia shows rates consistently lower than 10% [3]. In Saudi Arabia, a nationwide study showed a prevalence of 28.7% [4].

Several risk factors have been correlated with GERD by several studies. These factors include; dietary habits, tobacco smoking, obesity and stress [3,5,6].

Previous studies revealed that college students represent a vulnerable population that has a higher risk of GERD compared to general population [5,7]. This is probably due to the fact that college students are more exposed to certain GERD risk factors including stress and stress related behaviors [8,9]. However, correlating GERD with students perceived stress and academic study is still lacking.

The objectives of the current study were to determine the prevalence of GERD among students in health and non-health care colleges in southwestern Saudi Arabia and to explore its correlates with students’ perceived stress, and personal and academic characteristics.

## 2 Materials and Methods

### 2.1 Study design and setting

A cross-sectional study was carried out in both male and female campuses of King Khalid University (KKU) during the academic year 2018–2019. The University is located in Aseer region in the southwestern part of Saudi Arabia. The area of Aseer region is about 80.000 square kilometers occupied with more than 1.6 million people. The university comprise 5 health colleges and 24 non-health colleges with a total number of 60.312 male and female students in the academic year 2014–2015.

### 2.2 Target population, sample size and sampling method

Students in health and non-health care colleges in KKU were the target population. Sample size was estimated by using Epi info program version 7.2 with the anticipated prevalence of GERD symptoms among college students 23.8% [7], 95% confidence level and acceptable margin of error of 2.5%. The calculated cluster size was 1113 students. To account for the possibility of non-response, 1200 students were targeted from each of health and non-health care colleges.

All health care colleges were included in the study. They were colleges of medicine, pharmacy, dentistry, nursing and applied medical science. Five non-health care colleges were selected by random method. They were colleges of education, science, humanities, administrative and financial sciences, and languages and translation. Participants were selected through stratified cluster sampling technique. Within each college, students were stratified by academic level. Within each level a cluster (section or study group) was selected. When possible, all registered students within each cluster were included.

### 2.3 Study tool and data collection

A self-reported questionnaire was distributed personally by medical students-during their training in community medicine course-between the participants. The questionnaire includes the following sections: a) personal data such as, age sex, marital status, smoking status and family income; b) academic data which include, name of college, academic level and Grade point average (GPA); c) Arabic version of GERD questionnaire (GerdQ) [10]; d) Arabic version of Cohen's Perceived Stress Scale (PSS) [9]. All incomplete questionnaires were excluded. The response rate was 80% in health care colleges and about 94% in non-health care colleges. The only reason for non-response was lack of interest.

### 2.4 Arabic version of GERD questionnaire (GerdQ)

GerdQ is a valid questionnaire used to explore the probability of GERD. It is consisted of 6 questions as follow: four positive questions to assess GERD symptoms (heartburn, regurgitation, sleep disturbance related to heartburn and regurgitation and use of medications) and two negative questions (epigastric pain and nausea). Each item rated from 0 to 3 depending on the rate of symptoms over the previous week. GERD was detected with a total score value of 8 or more [10]. Score value of 8 or more plus total score value of 3 or more for the impact questions (sleep disturbance and use of medications) indicted GERD with impact on daily life. The Arabic version of GerdQ was developed and validated for use among Arabic speakers [11].

### 2.5 Data analysis

The gained data were entered, revised, and analyzed using SPSS, version 22 software package (IBM, North Castle, NY, USA). Grading of PSS into low, moderate and high was done according to Cohen's et al. [12] as the following: scores ranging from 0 to 13 was considered low stress; scores ranging from 14 to 26 was considered moderate stress; scores ranging from 27 to 40 was considered high perceived stress. Chi-square test was used to compare health and non-health care colleges regarding personal and academic characteristics, and prevalence of GERD and high perceived stress. Crude odds ratio (cOR) and adjusted odds ratio (aOR) were calculated using univariate and multivariable logistic regression analysis respectively. Their 95% confidence intervals (95% CIs) were used to identify significant factors associated with GERD among students. In the multivariable logistic regression model, the dependent variable was GERD status and the independent variables were personal, academic and high perceived stress variables. P- value of less than 0.05 was considered statistically significant.

## 3 Results

Ten colleges of King Khalid University (KKU) were included in the study with a total number of 2878 of students. All health care colleges were included in the study with a total number of 1228 of students. These include the colleges of medicine (353, 12.3%), pharmacy (253,8.8%), dentistry (159, 5.5%), nursing (160,5.6%) and applied medical science (303, 10.5%). Also, five non-health care colleges were included in the study with a total number of 1650 of students. They were colleges of education (347,12.1%), science (326, 11.3%), Humanities (308, 10.7%, administrative and financial sciences (346, 12.0%) and languages and translation (323, 11.2%). The age of participants ranged from 18 to 36 years, with average of 21.56 (SD 1.59) years. They were proportionally distributed across the different levels of health and non-health care colleges in KKU. Of the 2878 participants 52.8% were females.

Compared to students in health care colleges, students in non-health care colleges involved more males (56.1% vs. 35.2%), younger (≤20 years) students (31.0% vs. 21.3%), more married students (8.4% vs. 4.6%), students with unsatisfactory family income (8.7% vs. 3.5%), smokers (16.75 vs. 9.9%) and ex-smokers (9.5% vs. 4.4%) and students in 1st to 3rd academic years (67.2% vs. 45.4%). Therefore, the two groups of students were not matched regarding these basic personal characters (Table 1).

**Table 1:** Personal and academic characteristics of health and non-health care colleges students.

Factors	Total	Health care colleges	Non- health care colleges	P-value
	N=2878	N=1228	N=1650	
	n (%)	n (%)	n (%)	
**Age (years)**				
≤20	772 (26.8)	261 (21.3)	511 (31.0)	0.001
20	2106 (73.2)	967 (78.7)	1139 (69.0)	
**Sex**				0.001
Male	1358 (47.2)	432 (35.2)	926 (56.1)	
Female	1520 (52.8)	696 (64.8)	724 (43.9)	
**Marital status**				
Married	194 (6.7)	56 (4.6)	138 (8.4)	0.001
Single	2684 (93.3)	1172 (95.4)	1512 (91.6)	
**Smoking status**				
Never smoker	2271 (78.9)	1052 (85.7)	1219 (73.9)	0.001
Ex-smoker	210 (7.3)	54 (4.4)	156 (9.5)	
Current smoker	397 (13.8)	122 (9.9)	275 (16.7)	
**Family income**				
Satisfactory	2692 (93.5)	1185 (96.5)	1507 (91.3)	0.001
Unsatisfactory	186 (6.5)	43 (3.5)	143 (8.7)	
**Academic year**				
1-3	1665 (57.9)	557 (45.4)	1108 (67.2)	0.001
4-6	1213 (42.1)	671 (54.6)	542 (32.8)	
**GPA**^**a**^				
≤ 2.5	82 (3.6)	23 (2.7)	59 (4.1)	0.082
2.5	2192 (96.4)	823 (97.3)	95.9 (1369)	

GPA= Grade point average, ^a^ = missed data for 604 students

According to Table 2, GERD symptoms was detected among 955 students with a prevalence of 33.18%. Out of them 442 (46.3%) suffered GERD with impact on daily life with a prevalence of 17.2%. Overall GERD and GERD with high impact on daily life were significantly (p = 0.001) higher among students in non-health care colleges (36.6% and 19.2% respectively) compared to health care students (28.6% and 14.7% respectively). On the other hand, high perceived stress was detected among 364 students with a prevalence of 12.6%. This was significantly (p = 0.002) higher among health care students (14.4%) compared to non-health care students (11.3%).

**Table 2:** GERD and perceived stress in health and non-health care colleges students.

	Total	Health care Colleges	Non-health care Colleges	P- value
	n (%)	n (%)	n (%)	
Overall GERD	955 (33.2)	351 (28.6)	604 (36.6)	0.001
GERD with high impact on daily life	496 (17.2)	180 (14.7)	316 (19.2)	0.002
Low perceived stress	531 (18.5)	197 (16.0)	334 (20.2)	0.002
Moderate perceived stress	1983 (96.9)	854 (69.5)	1129 (68.4)	
High perceived stress	364 (12.6)	177 (14.4)	187 (11.3)	

GERD=gastroesophageal reflux disease

Table 3 shows the Univariate and multivariable analysis of factors associated with GERD among university students. Multivariable analysis showed that GERD was significantly (P-value <0.05) higher among male students (aOR = 1.44, 95% CI:117–1.65) compared to females. Similarly, the following factors were found significantly (p < 0.05) associated with GERD: being ex-smoker (aOR = 1.84, 95% CI: 1.33–2.54) or current smoker (aOR = 1.71, 95% CI: 1.31–2.23), being student in non-health care colleges (aOR = 1.36, 95% CI:1.12–1.65) and exposed to high perceived stress (aOR = 1.30, 95% CI: 1.01–1.44). On the other hand, no significant (P-value >0.05) relations were detected between GERD and academic level and GPA.

**Table 3:** Univariate and multivariable analysis of factors associated with GERD among study college students.

Factors	Total	GERD	cOR (95%CI)	aOR(95%CI)
	n (%)	n (%)		
**Overall**	2878(100)	955 (33.2)	-	-
**Age (years)**				
≤20	772	222 (28.8)	Ref	Ref
20	1846	733 (34.8)	1.32(1.10-1.58)*	1.17(0.92-1.47)
**Sex**				
Male	1358	551 (40.6)	1.52 (1.37-1.69)*	1.44 (1.17-1.65)*
Female	1520	404 (26.6)	Ref	Ref
**Marital status**				
Married	194	77(39.7)	1.21 (1.01-1.45)*	1.14 (0.68-1.89)
Single	2684	878 (32.7)	Ref	Ref
**Smoking status**				
Never smoker	2271	655(28.8)	Ref	Ref
Ex-smoker	210	106 (50.5)	2.51 (1.89-2.93)*	1.84 (1.33-2.54)*
Current smoker	397	194 (48.9)	2.36 (1.89-2.93)*	1.71(1.31-2.23)*
**Family income**				
Satisfactory	2692	861 (32.0)	Ref	Ref
Unsatisfactory	186	94 (50.5)	2.17(1.61-2.93)*	1.44(0.91-2.00)
**College**				
Non- health colleges	1650	604 (36.6)	1.44(1.23-1.69)*	1.36(1.12-1.65)*
Health colleges	1228	351 (28.6)	Ref	Ref
**Academic year**				
1-3	1665	544 (32.7)	Ref	Ref
4-6	1213	411 (33.9)	1.06 (0.90 – 1.23)	1.01 (0.82 – 1.24)
**GPA**^**a**^				
≤ 2.5	82	38 (46.3)	1.42(1.12-1.81)*	1.56 (0.85-2.94)
2.5	2192	715 (32.6)	Ref	Ref
**Perceived stress**				
Low to moderate	2514	814 (32.4)	Ref	Ref
High	364	141 (38.7)	1.32 (1.05-1.66)*	1.30 (1.01-1.44)*

GPA Grade point average, ^a^ = missed data for 604 students, ref = reference group, cOR= crude Odds Ratio, aOR=adjusted Odds Ratio, 95%CI= 95% confidence interval.

* P-value <0.05 (significant).

## 4 Discussion

To our knowledge, the present work is the first study evaluating the prevalence of GERD among young community of health and non-health care collages in the Southwestern Saudi Arabia. The study also, correlates GERD with some important determinants like personal and academic factors and perceived stress.

Our results show that GERD is a prevalent problem among college students as it affects about one third (33.2%) of the students. This figure is lower than the prevalence of 53.2% reported among college students in Western region of Saudi Arabia [13] but higher than the prevalence reported in Shaqra university (23.8%), Saudi Arabia [7]. Previous studies have revealed that college students may be at a greater risk of GERD when compared to general population [5]. Population based studies conducted among general population in two regions of Saudi Arabia showed a prevalence of 45.4% in Riyadh [4] and 23.4% in Western region [14]. Another nationwide study in Saudi Arabia revealed a prevalence of 28.7% [4]. Worldwide the prevalence of GERD show a wide variation and ranged from 2.5% to 7.8% in East Asia, 8.7%–33.1% in the Middle East, 8.8%–25.9% in Europe, 18.1%–27.8% in North America, 23.0% in South America and 11.6% in Australia [15].

In the current study, about one fifth of all students and nearly one half of the students with GERD have symptoms which affect their daily activity. Association between GERD and impaired quality of life has also been reported in subjects from Saudi Arabia [16], China [17] and North America [18]. In our study, considering the reported high prevalence of GERD, the associated daily life impacts, the young age of the studied subject and the risk of future complications, GERD among college students could represent a challenging health and economic problem. The economic load of the health care system related to GERD is caused by the costs of consultations, diagnosis, and management [19].

The current study observed that GERD was significantly higher among male students, ex-smokers and smokers, students in non-health care colleges and subjects exposed to high perceived stress.

Regarding that male students have a higher probability of acquiring GERD compared to females. This was in accordance with study conducted among university students in Shaqra Saudi Arabia [7]. However, controversy still existing about sex difference in GERD prevalence in population-based studies. While most of the studies reported no difference, a few studies found female predominance [7] and an Indian study found male predominance [20]. On the other hand, most of endoscopy-based studies have described a higher prevalence of GERD among men [21].

Regarding the elevated risk of GERD among smokers and ex-smokers, linking GERD with current and former tobacco smoking is evident in previous study [22]. Moreover, the benefits of smoking cessation in control of GERD symptoms have been also reported by previous studies [3,23].

The results of present study revealed a lower risk of GERD among health care students compared to non-health care students. This could be explained by the nature of their education which may positively influence their awareness and attitude toward GERD symptoms and possible risk factors [24]. Also, a previous study observed that medical college students may face a number of barriers in exhibiting or asking help for their health problems [25]. Another explanation in the light of the current study is related to the lower prevalence rates of current, and former smoking, and unsatisfactory family income among health care students compared to non-health care students. Smoking is a known risk factor for GERD [22] also, low income was found to be associated with some lifestyle factors, such as smoking and excess fatty diet intake which in turn could increase the risk of GERD [13,20,26].

In the present study, 12.6% of the college students suffered high level of perceived stress and the subjects exposed to high perceived stress at higher risk of GERD. Previous studies have shown a positive association between stress and reflux esophagitis [8,13]. Exposure to stress increases the secretion of gastric acid, slows and delays the gastric emptying, and causes the reflux [8]. Moreover, previous study reported that most of patients with GERD suffered exaggerated symptoms when faced stressful events [27].

### 4.1 Limitations

This is the first work in southwestern Saudi Arabia to investigate the prevalence of GERD and to correlate it with some important factors including academic study, smoking and stress among large sample of college students. However, the study has some limitations. As a cross-sectional design, the direction of the association between studied factors and GERD cannot be established. Also, some of potential risk factors of GERD as body mass index and dietary habits were not studied. Another limitation, the data were obtained by self-report questionnaire. Answers may have been altered by recall bias.

## 5 Conclusions

GERD is a prevalent problem among college students in southwestern Saudi Arabia as it affects about one third of the students. Moreover, one fifth of all students and nearly one half of the students with GERD have symptoms which affect their daily activity. Considering the high prevalence of GERD, the associated daily life impacts, the young age of the studied subject and the risk of future complications, this condition could represent a challenging health and economic problem. The risk of GERD is higher among; male students, smokers and former smokers, students in non-health care colleges and students exposed to high perceived stress. Developing smoking and stress management programs at universities could be beneficial in competing GERD.

## Ethical approval

The research has been approved by The Ethical Committee of the Scientific Research, King Khalid University (ECM#2019–23).

## Sources of funding for your research

No Funding.

## Author contribution

Study concept, design, data coding, entry, and analysis, writing the paper were done by the author.

Acknowledgment of data collection by the trained medical students added at the end of manuscript.

## Registration of research studies

Name of the registry: **ClinicalTrials.gov Protocol Registration and Results System (PRS**).

Unique Protocol ID: ECM.

ClinicalTrials.gov ID: NCT04034017.

## Guarantor

Author.

Awadalla NJ, Associate professor in Family and Community Medicine Department.

College of Medicine, King Khalid University, Abha, Saudi Arabia.

Tel: +966533487152, Fax: +9662418139.

## Provenance and peer review

Not commissioned, externally peer reviewed.

## Consent

During data collection, the data collectors introduced themselves to the participants in each grade and informed them about the study objectives and about warranties of anonymity and confidentiality of their data. The subjects’ participation was completely voluntary. The author confirms that the study was conducted according to the ethical standards of the relevant national and institutional committees on human research and with the Helsinki Declaration of 1975, as revised in 2008.

## Declaration of competing interest

No conflicts of interest.
